# Refugee and Migrant Women's Views of Antenatal Ultrasound on the Thai Burmese Border: A Mixed Methods Study

**DOI:** 10.1371/journal.pone.0034018

**Published:** 2012-04-13

**Authors:** Marcus J. Rijken, Mary Ellen Gilder, May Myo Thwin, Honey Moon Ladda Kajeechewa, Jacher Wiladphaingern, Khin Maung Lwin, Caroline Jones, François Nosten, Rose McGready

**Affiliations:** 1 Shoklo Malaria Research Unit, Mae Sot, Tak, Thailand; 2 Kenyan Medical Research Institute – Wellcome Trust Research Programme, Kilifi, Kenya; 3 Department of Public Health & Primary Care, University of Oxford, Oxford, United Kingdom; 4 Centre for Tropical Medicine, Nuffield Department of Clinical Medicine, John Radcliffe Hospital, University of Oxford, Oxford, United Kingdom; 5 Faculty of Tropical Medicine, Mahidol University, Bangkok, Thailand; UCL Institute of Child Health, University College London, United Kingdom

## Abstract

**Background:**

Antenatal ultrasound suits developing countries by virtue of its versatility, relatively low cost and safety, but little is known about women’s or local provider’s perspectives of this upcoming technology in such settings. This study was undertaken to better understand how routine obstetric ultrasound is experienced in a displaced Burmese population and identify barriers to its acceptance by local patients and providers.

**Methodology/Principal Findings:**

Qualitative (30 observations, 19 interviews, seven focus group discussions) and quantitative methods (questionnaire survey with 644 pregnant women) were used to provide a comprehensive understanding along four major themes: safety, emotions, information and communication, and unintended consequences of antenatal ultrasound in refugee and migrant clinics on the Thai Burmese border. One of the main concerns expressed by women was the danger of childbirth which they mainly attributed to fetal malposition. Both providers and patients recognized ultrasound as a technology improving the safety of pregnancy and delivery. A minority of patients experienced transitory shyness or anxiety before the ultrasound, but reported that these feelings could be ameliorated with improved patient information and staff communication. Unintended consequences of overuse and gender selective abortions in this population were not common.

**Conclusions/Significance:**

The results of this study are being used to improve local practice and allow development of explanatory materials for this population with low literacy. We strongly encourage facilities introducing new technology in resource poor settings to assess acceptability through similar inquiry.

## Introduction

Antenatal ultrasound has become part of standard antenatal care in the developed world[1]. This technology equally suits developing countries as well by virtue of its versatility, relatively low cost and safety[2]–[4] compared with other imaging modalities. In clinics in western Thailand, serving migrant workers and refugees from Burma, obstetric ultrasound has been adopted as part of routine antenatal care since 2001[5]. Yet it is not known how this technology is viewed by pregnant women, or by the local providers implementing the system. Recent literature highlights the usefulness of antenatal ultrasound in developing country settings[2], [6]–[8], but at the same time over-and misuse of ultrasound have been reported[9], [10].

Globally, not much is known about women’s or provider’s perspectives of obstetric ultrasound in low income settings. A systematic review of the literature on women's views of pregnancy ultrasound[11] identified one district hospital in Botswana[12], where ultrasound scanning was associated with significant psychological stress and anxiety in pregnant women, especially when accompanied by minimal explanation by healthcare providers. In different settings in Nigeria women were satisfied with most aspects of antenatal ultrasound experience[13], but incorrect determination of fetal sex had an important negative impact on women’s psychosocial health and general acceptance of antenatal ultrasound[14].

**Figure 1:**
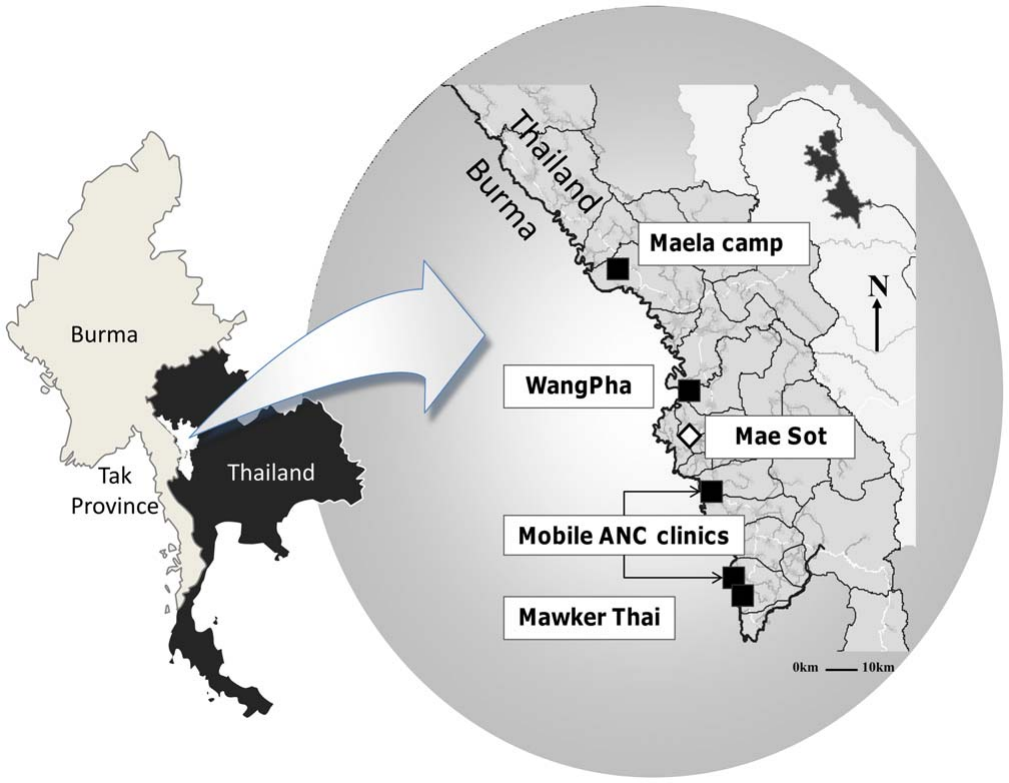
Geographical setting of the antenatal clinics of the Shoklo Malaria Research Unit. Location of the Shoklo Malaria Research Unit antenatal clinics and Mae Sot, the main town in the Thai province of Tak, bordering Burma. The locations of the antenatal clinics are represented by squares.

By contrast, in industrialized countries ultrasound scanning is associated with positive emotion: hope, reassurance and a sense of enhanced connection with the fetus[11], [15]–[18]. Most women appreciate seeing the image of the fetus and hearing verbal reassurance from the ultrasonographer[19]. This social component is so prominent that women may be unaware of the medical indications for the procedure and potentially unprepared for adverse findings[20].

This study was undertaken to better understand how routine obstetric ultrasound is experienced in a developing country setting, in particular in a displaced Burmese patient population. The results of this study are being used to improve local practice and allow development of explanatory materials for this population with low literacy[5], [21].

## Methods

### Background and study population

This investigation took place in the Shoklo Malaria Research Unit (SMRU) antenatal clinics (ANC) of Maela refugee camp (MLA), Mawker Thai (MKT), and WangPha (WPA), as well as two mobile clinics under supervision of MKT (see Figure 1). The SMRU is located on the Thai-Burmese border and has conducted research focused on the epidemiology, prevention and treatment of malaria in pregnancy since 1986. This has included provision of free obstetric and medical care for the local Burmese population, mostly of the Karen ethnic minority. The border population in this area consists of a mixture of Buddhist and Christian groups, with Muslims constituting a significant minority, more in the refugee than migrant communities. The refugee situation is one of the oldest in the world. As a low proportion of women could reliably provide the date of their last menstrual period [21], antenatal ultrasound was introduced in 2001 to improve gestational age estimation. Furthermore, ultrasound examination of the fetus is a powerful tool to detect multiple pregnancy, placental localization and intra-uterine growth restriction. Ten locally trained health workers perform ultrasound scans at all sites free of charge, supervised by doctors certified in ultrasound scanning[5].

### Ethics

This investigation was part of a larger fetal growth study (ClinicalTrials.gov Identifier: NCT00840502), and was approved by Oxford University (OxTREC (14–08)) and Mahidol University (TMEC 2008–028) Ethics Committees.

### Data collection

Qualitative (observations, interviews, focus group discussions (FGD) [22]) and quantitative methods (questionnaire survey) were used to provide a comprehensive understanding of the subject. The techniques were employed iteratively, with the results from one method feeding into the development of subsequent data collection tools, focused on four major themes: safety, emotions, information and communication, and unintended consequences of antenatal ultrasound.

Observations of ultrasound scans were used to develop a topic guide for semi-structured interviews with a selection of pregnant women. Native speakers (including authors MM and KML) conducted the interviews, which were recorded with the participants’ permission. The recordings were transcribed into English language and confirmed by a second interpreter. One author (MEG) interviewed experienced midwives who worked in the ANCs since before 2001 to elicit information on the impact of the introduction of ultrasound on midwifery practice. Subsequently, FGDs with providers (one group) and pregnant women (six groups stratified by language and religion) were organized to further investigate issues raised during the individual interviews. These were analyzed within the framework of the four themes. Finally, a questionnaire was designed to investigate whether the interviews and FGDs reflected pregnant women using the ANC services as a whole. Due to low literacy in this population[21], these were facilitated by local staff trained to obtain information anonymously and confidentially without suggesting responses. All women presenting to the ANC clinics over the course of a month were invited to complete the survey once, and women involved in the FGDs and interviews were excluded.

### Statistical analysis

The results of the questionnaires were entered into a Microsoft Access database and analyzed using SPSS version 18 (SPSS Inc., Chicago Ill, USA). Student’s t-test and Mann-Whitney test were used for comparison of means and ranks respectively. Categorical data were compared using the chi-squared test or the Fisher’s exact test, as appropriate, with Bonferroni correction in case of multiple comparisons.

## Results

Between November 2010 and February 2011, 30 ultrasound scans were observed and 19 interviews were conducted; 17 with pregnant women and two with senior midwives. The seven FGDs included one with four sonographers, three with six Christian, Buddhist and Muslim women each, two with Karen (six women) and Burmese (seven women) from mixed religious backgrounds, and a mixed group of six participants. The discussions lasted from 30 minutes to an hour. The questionnaire (See file S1) was completed by 67% (644/964) women who attended the ANC and were eligible (Table 1).

**Table 1:** Demographics of 644 women participating in the questionnaire.

Woman’s age, years		26.0 [15–47]
Woman’s marriage number		1 [1]–[3]
Husband’s age, years		28.5 [17–65]
Husbands marriage, number		1 [1]–[4]
Number of pregnancies		2 [1]–[12]
Parity (number of delivered infants)		1 [1]–[9]
Residence on the Thai-Burmese Border, months		48 [0–576]
Schooling, years		4 [0–16]
Previous ultrasound scans		2 [0–13]
Location	*MaeLa refugee camp*	53.7 (346)
	*Mawker Thai*	15.7 (101)
	*Wang Pha*	30.6 (197)
Teenager		14.8 (95)
Reports ability to read		64.4 (415)
Reports ability to write		64.0 (412)
Religion	*Buddhist*	69.6 (448)
	*Christian*	21.0 (135)
	*Muslim*	9.3 (60)

Data are in median [range], or percentage (number).

### Safety

#### Safe Pregnancy and Delivery

Forty-one percent of the interviewed women (7/17) highlighted the danger of pregnancy, when asked about the usefulness of ultrasound. Women were primarily concerned about how antenatal care and the use of ultrasound could increase the safety of what they see as a potentially life threatening event of childbirth.

***“I came to SMRU because pregnancy is dangerous… I came for safety and deliver here. Home delivery is not safe. Before ultrasounds, women would deliver in the village and they wouldn’t know the baby’s position. Because they might try to deliver a baby that was in the wrong position in the village, they would have serious problems with bleeding and other things” [23 yo Karen G1 at WPA].***

The most common concern noted in the interviews and FGDs was the position of the fetus. Other safety concerns mentioned included bleeding, premature delivery, multiple pregnancies (twins), and miscarriage. A 38-year old woman in MLA stated:

***"I have had many pregnancies so I am afraid of complications. If tharamu*** [word of respect for someone knowledgeable e.g teacher, midwife] ***does the ultrasound then she can detect problems ahead of time, and maybe she can even save my life."***

In addition to fetal position, the experienced midwives highlighted early pregnancy bleeding and antepartum hemorrhage as examples of potential obstetric emergencies, where ultrasound had improved practice safety and decreased the need for referral:

***“Before the ultrasound, if someone came in with early pregnancy bleeding we could not do a dilatation and curettage because we did not know about if there was a fetal heartbeat or not. With ultrasound now we can know the presentation, the location of the placenta, about any fetal abnormalities, and about the fluid level. Before ultrasound we estimated based on the clinical exam but we can know more with ultrasound. For example, before if there was antepartum hemorrhage we might not be sure if just close to delivery, or if it was placenta praevia. Before, we would refer all women with antepartum haemorrhage to the hospital, but with the ultrasound we can check and only refer if there is an indication.”***

***[45 year old midwife with 25 years experience]***

The greater certainty in diagnosis and therefore improved safety for patients convinced both midwives that antenatal ultrasound is beneficial. In the group discussion with the sonographers, who are mostly unmarried and younger women, safety was not raised as a primary concern. They expressed increased personal interest, but also some distress, when abnormal findings were found.

In an open ended question in the survey, determination of fetal position was the most commonly named reason for ultrasound (Table 2). These results, however, differed somewhat by site, with the majority of patients at MLA and one of the mobile sites reporting gender determination most frequently as the reason for performing the ultrasound.

**Table 2:** Responses to open questions in a questionnaire among 644 pregnant women on the Thai Burmese border.

Why does SMRU do ultrasound scanning for pregnant women?	% (N) 1696 answers
*Position of the baby*	22.1 (375)
*Confirmation of pregnancy*	20.2 (343)
*Health of the baby*	18.8 (318)
*Sex of the baby*	17.3 (293)
*Normal hands and feet*	8.7 (148)
*Baby's breathing*	6.8 (116)
At the most recent scan, what did they explain before starting?	% (N) 1414 answers
*I would need to lie down*	40.2 (569)
*I would need to open my sarong*	23.1 (326)
*A machine would be used to check the baby*	14.8 (209)
During or after the most recent ultrasound, what did they tell you?	% (N) 1224 answers
*Everything is okay*	24.5 (300)
*They told me the sex of the baby*	19.9 (243)
*They told me the position*	16.3 (200)
*They told me that I am pregnant*	10.5 (129)
*Nothing*	8.6 (105)

This table refers to questions 6–8 of the questionnaire in file S1.

#### Abnormal findings

The interviewed midwives raised the concern that women may discontinue antenatal care after abnormal results found by ultrasound are given to them. Such women sometimes go to traditional birth attendants (TBAs) for treatments or to seek unsafe abortions. One example is that of a woman who learns that the fetus is in breech position. If there were no contraindications she would routinely be scheduled for an external cephalic version – a process of rotating the near-term fetus using external pressure on the abdomen, while monitoring the fetal wellbeing with ultrasound. In the clinic this is always performed by a physician and only if there is an emergency car available for transport to a referral hospital in case of complications. However, TBAs in the community also provide this service, sometimes with tragic results. In the surveys, 6.2% (40/644) of respondents reported they would seek care with a TBA in addition to continuing care at the SMRU clinic if told the fetus was breech. These responses were independent of parity but were more common among Buddhist patients (8.3%) compared to Muslim (3.3%) and Christian (0.7%), the latter being significantly different, p = 0.021 (Table 3). There was a trend toward higher frequency in TBA visits in illiterate patients; of concern one illiterate Buddhist multiparous woman reported that she would seek care with a TBA only in this situation, and not with the SMRU clinic. If the fetus was found to be “abnormal” by ultrasound 3.1% (20/644) of women reported they would seek care with a TBA in addition to SMRU, and 1.7% (11/644) would do so if there were no fetal heartbeat found (Table 3). There was no deeper questioning about why these choices would be made.

**Table 3:** Responses to “what would you do if?” questions in a questionnaire among 644 pregnant women on the Thai Burmese border.

	Multi	Primi	Cannot read	Can Read	Many US	First US	Buddhist	Christian	Muslim
	n = 440	n = 203	n = 217	n = 415	n = 441	n = 202	n = 447	n = 135	n = 60
**What would do you in the breech presentation?**									
Continue normal ANC	411	191	201	390	412	190	409	134	58
ANC+ TBA	28	12	15	25	28	12	37	1	2
TBA	1	0	1	0	1	0	1	0	0
I do not know	0	0	0	0	0	0	0	0	0
Other	0	0	0	0	0	0	0	0	0
**What would do you if the ultrasound tells you your baby is abnormal?**									
Continue normal ANC	427	196	212	400	428	195	430	132	60
ANC+ TBA	13	7	5	15	13	7	17	3	0
TBA	0	0	0	0	0	0	0	0	0
I do not know	0	0	0	0	0	0	0	0	0
Other	0	0	0	0	0	0	0	0	0
**What would do you if the ultrasound tells you your baby has no Fetal Heart Beat?**									
Continue normal ANC	433	196	213	405	434	195	433	135	60
ANC+ TBA	6	5	4	7	6	5	11	0	0
TBA	0	0	0	0	0	0	0	0	0
I do not know	0	0	0	0	0	0	0	0	0
Other	0	0	0	0	0	0	0	0	0
**What would do you if the ultrasound tells you are pregnant, but you do not want this pregnancy?**									
Continue normal ANC	403	188	201	379	405	186	410	127	53
ANC+ TBA	29	8	13	24	25	12	34	2	1
TBA	7	4	3	8	8	3	1	4	6
I do not know	1	1	0	2	2	0	1	1	0
Other	0	0	0	0	0	0	0	0	0

This table refers to questions 14–17 of the questionnaire in file S1.

#### Safety of the Ultrasound scan

Women were almost unanimous in reporting that they felt ultrasound scanning was safe to them and their babies. This confidence was attributed both in the interviews and the FGDs to faith in the providers at the clinic:

***“If tharamu says there is no problem, then I think there is no problem. If there were a problem, she would tell me. So I am not worried” [29 yo Burmese G1].***

After the official discussion in one FGD, a pregnant medic asked if there were any risks to repeated scans. She referred to a rumor in MLA that ultrasound could damage the fetal brain, but was not sure whether the ultrasound scan were performed because of a brain abnormality. The sonographers reported that Burmese patients expressed more concern about safety of the ultrasound than Karen patients, but that all patients appeared satisfied with some reassurance.

***“Some patients think if we do the ultrasound frequently then there will be some danger to the baby.” [23 year old sonographer, 4 years experience]***

In the surveys, 5.1% (33/644) of respondents reported that they believed it could be dangerous, with no differences between gravidity or religion.

### Emotions: Shyness and anxiety

Experiences of shyness and anxiety were noted during the observations and were themes that emerged in the interviews. In each room one sonographer and two other staff members engaged in interviewing patients were present with the pregnant women, who occasionally brought small children into the room. Usually another pregnant woman was already waiting inside the room as well. In ultrasound rooms that were not fully enclosed and private, women showed body language consistent with discomfort–squirming, attempting to cover the belly etc. In this community, where breastfeeding in public is accepted, the abdomen is treated with particular modesty. “Showing the belly” was commonly mentioned in the interviews and FGDs as a notable part of prenatal care.

The sonographers reported that some women try to cover their abdomen before the scan is completed, and that this embarrassment was more common in younger women. On the other hand, pregnant women that received multiple scans appeared relaxed, even bored.

When the emotional impact of the ultrasound was initially probed in the interviews by asking the women to describe what happened at their first ANC visit, 71% (12/17) of the women did not mention the ultrasound at all. Positive and neutral feelings by far exceeded negative feelings and many seemed to include it as part of the routine obstetric exam. Women used terms conveying, “It was no big deal, it was no problem.” Women expressed that they were “happy”, often in the context of relief to know that the pregnancy is confirmed, that the fetal position was correct and that the baby appeared alive and normal. A minority of women expressed negative emotions that seemed to reflect the discomfort noted in the ultrasound observation.

***“I am a little embarrassed about the pregnancy because they uncovered my belly. So I am a little shy” [29 yo Burmese G1 from MKT].***

Another stated she felt

***“Ashamed because it was in front of all the other pregnant women” [28 yo Burmese G3P2].***

when presenting for her first ultrasound at a migrant clinic where the scan is done in the general waiting area. However, when probed further she said she would have still felt shy even if the scan was done in private. Women in the FGDs reported that this shyness or shame disappeared completely by the second ultrasound. All agreed that it was not a problem for a male healthcare provider to enter the room when needed. In the FGDs, embarrassment was reported most strongly in the Muslim group (4/6), followed by the Buddhist (2/6), and Christian group (0/6). The questionnaire showed a significantly higher prevalence of embarrassment among primiparous women (18.2% (37/203)) compared to multigravidae (11.6% (51/440)), p = 0.023, those experiencing their first ultrasound (17.8 (36/202)) vs many ultrasound scans (11.8% (52/441)), p = 0.039, and Muslim religion (28.3% (17/60) vs Christian (6.7% (9/135)), p<0.001, but there was no significant difference in shyness based on education or literacy (table S1). Women reported a greater degree of shyness at WPA (19.7% (39/197)), where scans were done in the semi-private room, than in MLA (11.3% (39/345)), where the ultrasound room is private (closed wooden door, high walls), p = 0.032

The second negative emotion expressed was a sense of anxious anticipation:

***“my heart was racing… because I have never had an experience with this machine before” [19 yo G1 Karen at MKT].***

In the individual interviews, this was exclusively expressed by women presenting for their first ultrasound. Women in the FGD and the individual interviews stated that this feeling disappeared immediately after the ultrasound scan started and was not present for the second ultrasound. In the questionnaire, this anxiety was more common at MLA with the private ultrasound room, where women cannot see what happens to the women who went ahead of her (26.1% (90/345) compared to WPA (17.8% (35/197)), p = 0.027. Women in the FGD stated that provision of further information prior to the ultrasound would greatly reduce this anxiety.

Though the questionnaire confirmed a decrease in embarrassment and anxiety with greater experience, it did not corroborate the consensus of the FGDs and individual interviews that these emotions were confined to the initial ultrasound experience. Among veteran ultrasound users, 11.8% (52/441) reported shyness at their most recent ultrasound and 20.6% (91/441) reported anxiety. As with embarrassment, anxiety levels differed by religious group: most commonly reported by Muslim women (50.0% (30/60)), followed by Christian (26.7% (36/135)) and Buddhist (17.7% (79/447)), p<0.001.

### Information and Communication

During the ultrasound observations, minimal sonographer communication with patients was noted. All sonographers were bi- or tri-lingual (Karen, Burmese and in most cases English language) but they frequently chatted in their primary language with one another. Patients who shared the same primary language sometimes joined these conversations. Women of other language groups lay in silence or, rarely, asked a question.

Overall, the counseling varied depending on the indication for the ultrasound. For all patients presenting for their first ultrasound, prior history and risk factors were reviewed as routine practice. No major differences were observed among the sonographers. Minimal explanation was given to women having their dating or routine biometry scan, although such scans could take 30 minutes or more. At the other extreme, scans for placenta position lasted less than five minutes but, in cases of low-lying placenta (two of 30 scans observed), were accompanied by concurrent counseling that exceeded the time spent performing the scan. In the one case observed in which there were catastrophic findings – no fetal heart beat at term – minimal explanation was given to the patient until a midwife was asked for help.

Any counseling about ultrasound process or results, reported in the interviews and FGDs, was minimal, and generally restricted to friendly spoken directives: “lie down, open your sarong” etc:

***“They told me that the baby is well and the position is okay, and then counseled me about what to avoid in pregnancy and other things” [29 yo G1 Burmese at MKT].***

This was supported by the 1,414 answers to the question “At your most recent US, what did the staff explain to you before they started to scan?”. The most frequent answers in all sites were: “I would need to lie down” (88.4% (569/644)), “I would need to open my sarong” (50.6 % (326/644)) and “a machine would be used to check the baby” (32.5% (209/644)). The third most common response at WPA was “it is safe” (30.5% (60/197)) and more than 20% of the women at the two mobile ANC sites reported they were told “nothing” before the test.

As noted above, women in the interviews and FGDs expressed knowledge that the ultrasound was used to detect potential problems for delivery (malpresentation, twins), confirmation of pregnancy, checking for fetal health (“breathing”, movement, if the baby is strong or not), ruling out abnormal development (normal hands and feet) and determining gender. The questionnaire showed a variety of responses both for content of post ultrasound counseling and understanding of the reasons for the test (Table 2). Women at MLA reported most often being counseled about fetal sex and reported this most often as the reason for the scan, while women at WPA reported fetal position as the primary reason for the test. This suggested that, even in this setting of minimal counseling, patients did internalize as significant the information they received.

Sonographers felt that time pressure, due to patient volume, limits their ability to give counseling beyond the essentials. They noted that women rarely ask questions. When they do, the most common questions are about gender, position, fetal heart beat and whether or not the infant was normal.

Patient satisfaction with levels of communication was probed in the FGDs. Most women expressed receiving some feedback about the scan, often “everything is okay”, and this was felt to be sufficient. General statements from the sonographer about fetal health, position and gender were most commonly reported. A minority of women reported asking about these topics. Several women reported not receiving any counseling at all. Though some were content with this, others expressed continued curiosity and desire for further counseling. Others expressed having received detailed counseling about one scan, which they appreciated and this single episode of education seemed to satisfy them for future scans as well.

All women in the FGDs said that they would have liked to see the fetal image on the screen, with the exception of the Muslim group and one teenager in the mixed group. Most women in the interviews and FGDs stated that they could not see, or didn't know what they were looking at.

***“I saw small spots running around the screen” [28 yo Burmese G3P2 at WPA].***

Only two women in the FGDs reported that the sonographer showed her images of fetus and explained what was happening with it. Both women expressed very positive feelings about that experience, even though, for one it was in the setting of a miscarriage. One other woman reported that, though she couldn't see her own fetus on the screen, she recognized someone else's fetus while she was waiting in the room.

Overall, 90.4% (582/644) of women answered that they wished to see the screen, and 39.4% (254/644) reported that they were able to see it (table S1). Due to space considerations, the ease with which patients are able to see the screen differs significantly by site. The desire to see the screen was slightly higher in non-Buddhists, experienced patients and those who were literate. Interest was lowest in MKT (64.9 (24/37)) and the mobile sites (51.2 (21/41)), but above 90% in the larger clinics (MLA 98.0%, WPA 94.9%).

Another special topic of information sharing was sex determination. As in the developed world, many women in this community enjoy knowing the gender of their unborn child.

***“I think it is good to know the gender so you can prepare in advance; so you can dream for the future.” [23 yo Karen primigravid WPA]***

The ultrasonographers noted that gender was the most commonly asked question, and that they told patients, “when we remember”. Some sonographers admitted to sometimes rescanning women who were really curious about gender later in the day, after the regular scans were complete. Desire to know gender was reported by almost all participants (98.4% (634/643)) but disclosure differed markedly by site: 22.8% (45/197) in WPA and 53.5% (185/345) in MLA, p<0.001.

### Unintended Consequences: Gender Selection and Overuse

Located in Asia where gender selective practices are common[23], [24], questions were raised at all levels of the study to assess the risk of unintentionally facilitating gender selective abortion by introducing ultrasound. Unlike other populations, a preference for males is not as strongly held in this community, so the inquiry included termination of any pregnancy due to non-desired gender. As noted above, almost all participants expressed a desire to know the gender of the fetus. When asked directly in the interview setting, none of the participants expressed an intention to seek an abortion if told that they are carrying the less-desired gender.

***“If it is a girl, I want a girl. If it is a boy, I want a boy” [21 yo Karen G2P1 at MKT].***

***“No, I would not think of [an abortion], it is my own flesh and blood” [25 yo Karen G3P2 at MLA].***

Three women responded that they had heard of gender selective abortion in their communities. The experienced midwives expressed that they had seen many women present for care after unsafe abortion, but had not heard of this practice for gender selection. All FGDs reported knowing of abortions in their community, but that these were almost always for unwanted pregnancies in general, regardless of gender, and usually occurred before gender was known. While disapproving abortion in general, and gender selection specifically, women in the FGDs reported that this is an uncommon occurrence. Only 0.6% (4/644) respondents reported that they had heard of women seeking abortion after learning from an SMRU ultrasound that they are carrying a child of the undesired gender. Both in the FGD and in the questionnaire, Muslim women reported that no abortions are attempted for any reason in their community, which does not reflect clinical experience (unpublished data).

Special attention was paid to determine what impact the presence of antenatal ultrasound has on care-seeking behavior in this patient population. None of the women in the individual interviews named the ultrasound as a primary reason for seeking care at SMRU’s ANCs. The report by the sonographers that they would occasionally repeat or extend a scan to look for gender, suggests that there may be a risk for patient demand for ultrasounds, but at this point such demand appears to be low.

## Discussion

Qualitative studies on obstetric ultrasound in the developed world have focused on feelings of expectation, possibility, enhanced bonding (both maternal and paternal) with the fetus, and concern about the possibility of fetal anomaly[19]. One of the main concerns of women in this study was the danger of childbirth which they mainly attributed to fetal position. This correlates with the objective risk of pregnancy in this area: maternal and neonatal mortality in developing countries may be over hundred times higher than in western countries[25] (McGready, submitted). One of the top priorities of the Millennium Development Goals is to reduce maternal mortality. A large number of maternal deaths are caused by conditions that could be prevented or managed with the assistance of ultrasound, such as fetal malposition[7].

Although happiness was an emotion frequently endorsed by patients, transient embarrassment or shame on exposing the abdomen (a part not normally exposed in public by local women in this culture) was noted by primigravids or teenagers. Anxiety and “racing heart” was also reported in a few cases and appeared to be related to not knowing what kind of examination would occur and how it would be done. Further training in counseling for ANC and ultrasound staff and provision of simple tools to help them with patient education has the potential to alleviate distress and improve patient health knowledge.

In contrast with the profound fears about harm from the ultrasound scan due to factors as a dark examining room, foreign technicians and almost total language barriers as reported in Botswana[12], in this study no woman reported fears that an ultrasound scan was dangerous to themselves or to the fetus. This may be due to several reasons, firstly in the SMRU clinics these factors are not present, though at times a foreign doctor may perform part of a complicated scan, or the sonographers may discuss results in a language that the patient does not understand (eg. Karen for a Burmese patient). However, in this multilingual area, this is typical of daily life and not confined to the clinics. Secondly, pregnant women expressed an immense trust in the health providers, which may be due to the long existence of the SMRU ANC (25 years) or the method of frequently intermittent screening for malaria in which women are invited to come every week, and this inevitably results in a personal friendly attitude towards women who come regularly. Similar to the Botswana site was the paucity of patient counseling, frequently limited to “everything is okay”.

The participants and medical staff in this study overwhelmingly reported that they believe antenatal ultrasound improves patient safety and they would not want to have ultrasound services stopped. On the other hand, provider-driven overuse is unlikely to happen, mainly since there is no financial incentive for the providers to increase the number of scans[20].

Given the prevalence of gender selection in nearby countries[23], this study investigated carefully the potential unintended harm of antenatal ultrasound by determining gender prior to delivery. Though several women reported that they had heard of gender selective abortion, and women do seek unsafe abortions after confirming pregnancy by ultrasound, all denied any intentions of selecting for gender. There are many challenges to gathering this sensitive information, but these were minimized as much as possible by the mixed methods approach of our study. Seeking abortion after pregnancy confirmation occurred before the introduction of ultrasound and abortion rates did not show a significant change before and after ultrasound (unpublished data). Protective factors in the local culture may include a kind of fatalism rooted in the animist beliefs that pervade most peoples' world views.

### Limitations and future research

This study benefits from a mixed methods approach, drawing from both quantitative and qualitative techniques. However, more could be done in either of these research traditions–both filling out the qualitative description of the reception of ultrasound among the local cultural groups, and widening the scope of the quantitative investigations to include more aspects of the ultrasound experience. A more systematic observational study might be able to better quantify what counseling is routinely given, without relying on the participants’ memory. Due to time constrains in the busy antenatal clinics the local staff was able to complete the questionnaire in 67% of the eligible women, and this may have introduced some selection bias.

The disinterest in viewing the screen expressed by the participants in the Muslim FGD was anomalous and contradicted by the 96.7% of Muslim survey participants who reported interest in seeing the screen. This confirmed the impression held by those conducting the discussion that the results of that particular FGD were skewed by one outspoken older women whose voice dominated parts of the discussion. This dynamic was not observed in the other focus groups, where most of the opinions expressed were confirmed by the survey results.

While the main interviewer and leader of FGD (MM) is not an obstetric provider, she is a SMRU employee, and this may have affected the information the women were willing to reveal or the way in which they responded to questions. The SMRU has a long relationship with the communities in which it works, and they may be hesitant to give negative reports of their care. Respect for authority was evident in various answers we received, and this is a strong current in Burmese and Karen culture. Nevertheless, participants did report negative experiences in both group and private sampling settings, a fact that implies that these cultural barriers were not insurmountable.

### Implications for clinical practice

Changes within the clinic have already occurred based on these results including a brief explanation to all women about their first and future ANC visits by the enrolling midwife and including antenatal ultrasound in a health promotion video for pregnant women. The ultrasound rooms have been modified to allow more easy vision of the screen by the woman. The sonographers have had a workshop including role plays and focusing on greeting the woman and explaining what they will do, as well as inviting the woman to ask questions. However there are cultural and educational limits to what can be overcome.

The small number of women who reported they would see a TBA if there a problem reported is of concern and efforts have been made to counsel women receiving abnormal results about the dangers of such treatments. Patients are also encouraged to bring TBAs to the clinic for joint discussion of care, and collaboration, rather than seeking independent treatments concurrently. Another limitation of these data is that the setting of the questionnaire in the clinic could have introduced bias. It is unknown what percentage of women routinely seeks “double care” with TBAs in addition to SMRU but it has been normal for centuries to deliver with a TBA in this area.

Implementation of technological innovations in a resource poor setting is often initiated by outsiders and patient mistrust or discomfort can compromise otherwise well designed programs. Because of this, we would strongly advocate inquiry along similar lines to be done in other settings concurrent with the introduction of ultrasound in order to facilitate development of effective and acceptable programs.

## Supporting Information

File S1 Questionnaire (PDF)

Table S1 **Responses to defined questions (yes/no) in a questionnaire among 644 pregnant women on the Thai Burmese border.**
(DOC)
