# Supplementary material for: Refugee and Migrant Women's Views of Antenatal Ultrasound on the Thai Burmese Border: A Mixed Methods Study
Source: PLoS One. 2012 Apr 13;7(4):e34018. doi: 10.1371/journal.pone.0034018 (PMC3325974; doi:10.1371/journal.pone.0034018)
Supplement: File S1 Questionnaire — (PDF) [file pone.0034018.s001.pdf]

Ultrasound Questionnaire

Date: |__|__|-|__|__|-|__|__| ANC code: |__|__|__|- |__|__|__|__|__|

1. Religion[circle]: Buddhist / Christian / Muslim / Other: __________

2. How many ultrasounds (US) have you had? |__|__|

3. Years at school |__|__| years

4. Grade |__|__|

5. Other __________________________

---

6. Why do we do US for pregnant women? More answers are possible for question 6-8. If you need more space to write please turn page.

|__| **Position**[1]
|__| **Sex**[2]
|__| **Normal hands and feet**[3]
|__| **Health of the baby**[4]
|__| **Baby's breathing**[5]
|__| **Confirm pregnancy**[6]
|__| **How many babies**[7]
|__| **Other**[8]: ___________________________________
|__| **I don't know**[9]

7. At your most recent US, what did the staff explain to you before they started to scan?

|__| **Nothing**[1]
|__| **A machine would be used to check the baby**[2]
|__| **I would need to lie down**[3]
|__| **I would need to open my sarong**[4]
|__| **Not to worry**[5]
|__| **It is safe**[6]
|__| **The reasons for the US**[7]
|__| **Other**[8]:___________________________________
|__| **I don't know/I cannot remember**[9]

8. During or after the most recent US scan, what did they tell you?

|__| **Nothing**[1]
|__| **They explained what they were looking at**[2]
|__| **They said "Everything is okay"**[3]
|__| **They told me that I am pregnant**[4]
|__| **They told me the sex of the baby**[5]
|__| **They told me the position**[6]
|__| **They told me about a problem**[7]
|__| **Other**[8]:___________________________________
|__| **I don't know/I cannot remember**[9]

During your last scan: Please enter 1 answer only for question 9-21. If you need more space to write please turn page.

9. Did you feel shy? |__| **Yes**[1] |__| **No**[2]

10. Did you feel your heart racing or anxiety? |__| **Yes**[1] |__| **No**[2]

11. Did you think it was dangerous? |__| **Yes**[1] |__| **No**[2] |__| **I don't know**[3] |__| **No answer**[4]

12a.Did you see the screen? |__|**Yes**[1] |__|**No**[2] |__|**No answer**[3]

12b. Did you want? |__| **Yes**[1]|__| **No**[2]|__| **I don't care**[3]

13a.Did they tell the sex? |__|**Yes**[1] |__|**No**[2] |__| **No answer**[3]

13b. Did you want? |__| **Yes**[1]|__| **No**[2]|__| **I don't care**[3]

Write down 1,2,3 or 4 in the box to answer questions 14-17:

|_1_| **Continue with normal antenatal care**[1]
|_2_| **Continue with normal ANC but also go to TBA**[2]
|_3_| **Stop coming for antenatal care; only go to TBA**[3]
|_4_| **I don't know**[4]

14. What will you do if the US finds the baby is breech? |__| other[5]_____________________________
15. What will you do if the US finds the baby is not normal? |__| other[5]_____________________________
16. What will you do if the US finds the baby has no heart beat? |__| other[5]_____________________________
17. What will you do if the US confirms you are pregnant but you do not want to be pregnant? |__| other[5]_____

---

18. Have you ever heard of someone coming to SMRU for US and learning from the staff that the baby will be the sex that they do *not* want, and trying to get an abortion for that reason? |__| **Yes**[1] |__| **No**[2] |__| **No answer**[3]

19. Do you think US for pregnant women is useful? |__| **Yes**[1] |__| **No**[2] |__| **No answer**[3]

20. In SMRU US is free, at other hospitals it costs 500 baht. Could you pay that? |__| **Yes**[1] |__| **No**[2] |__| **No answer**[3]

21. Should we continue to do US in pregnancy? |__| **Yes**[1] |__| **No**[2] |__| **No answer**[3]

Thank you!

Interviewer Name ____________________________
