# Supplementary material for: Refugee and Migrant Women's Views of Antenatal Ultrasound on the Thai Burmese Border: A Mixed Methods Study
Source: PLoS One. 2012 Apr 13;7(4):e34018. doi: 10.1371/journal.pone.0034018 (PMC3325974; doi:10.1371/journal.pone.0034018)
Supplement: Table S1 — Responses to defined questions (yes/no) in a questionnaire among 644 pregnant women on the Thai Burmese border. (DOC) [file pone.0034018.s002.doc]

Supporting table S1

Responses to defined questions (yes/no) in a questionnaire among 644 pregnant women on the Thai Burmese border

| **Question number and topic** | | **Gravidity** | | **Literacy** | | **How many scans?** | | **Religion** | | |
| --- | --- | --- | --- | --- | --- | --- | --- | --- | --- | --- |
| **Subgroup** | | **Multi** | **Primi** | **Cannot read** | **Can Read** | **Many US** | **First US** | **Buddhist** | **Christian** | **Muslim** |
| **Number of women** | | **n=440** | **n=203** | **n=217** | **n=415** | **n=441** | **n=202** | **n=447** | **n=135** | **n=60** |
| **9. Shy?** | **Yes** | 11.6 (51) | 18.2 (37) | 14.7 (32) | 13.0 (54) | 11.8 (52) | 17.8 (36) | 13.9 (62) | 6.7 (9) | 28.3 (17) |
|  | **No** | 88.4 (389) | 81.8 (166) | 85.3 (185) | 87.0 (361) | 88.2 (389) | 82.2 (166) | 86.1(385) | 93.3 (126) | 71.7 (43) |
| **10. Anxiety?** | **Yes** | 21.4 (94) | 25.6 (52) | 18.9 (41) | 24.3 (101) | 20.6 (91) | 27.2 (55) | 17.7 (79) | 26.7 (36) | 50.0 (30) |
|  | **No** | 78.6 (346) | 74.4 (151) | 81.1 (176) | 75.7 (314) | 79.4 (350) | 72.8 (147) | 82.3 (368) | 73.3 (99) | 50.0 (30) |
| **11. Dangerous?** | **Yes** | 4.8 (21) | 5.9 (12) | 5.1 (11) | 5.3 (22) | 4.8 (21) | 5.9 (12) | 5.6 (25) | 4.4 (6) | 3.3 (2) |
|  | **No** | 94.5 (413) | 91.1 (185) | 93.5 (202) | 93.5 (386) | 94.3 (413) | 91.6 (185) | 92.4 (413) | 93.3 (126) | 96.7 (58) |
| **12a. See the screen?** | **Yes** | 38.4 (169) | 41.9 (85) | 35.5 (77) | 41.2 (171) | 42.6 (188) | 32.7 (66) | 31.5 (141) | 60.0 (81) | 53.3 (32) |
|  | **No** | 61.6 (271) | 57.6 (117) | 64.5 (140) | 58.6 (243) | 57.4 (253) | 66.8 (135) | 68.2 (305) | 40.0 (54) | 46.7 (28) |
| **12b. Want to see screen?** | **Yes** | 90.6 (397) | 91.1 (185) | 88.9 (192) | 91.5 (379) | 92.7 (407) | 86.6 (175) | 88.1 (394) | 96.3 (130) | 96.7 (58) |
|  | **No** | 9.1 (40) | 8.9 (18) | 11.1 (24) | 8.2 (34) | 7.3 (32) | 12.9 (26) | 11.4 (51) | 3.0 (4) | 3.3 (2) |
| **13a. Tell the sex?** | **Yes** | 40.2 (177) | 43.3 (88) | 33.5 (73) | 44.8 (186) | 46.3 (204) | 30.2 (61) | 34.7 (155) | 59.3 (80) | 50.0 (30) |
|  | **No** | 58.9 (259) | 56.2 (114) | 65.9 (143) | 54.5 (226) | 53.1 (234) | 68.8 (139) | 64.7 (289 ) | 40.0 (54) | 48.3 (29) |
| **13b. Want to know sex?** | **Yes** | 98.9 (435) | 98 (199) | 98.2 (213) | 99.0 (411) | 98.4 (434) | 99.0 (200) | 98.7 (442) | 29.5 (132) | 98.3 (59) |
|  | **No** | 0.9 (4) | 2.0 (4) | 1.4 (3) | 1.0 (4) | 1.4 (6) | 1.0 (2) | 1.1 (5) | 1.5 (2) | 1.7 (1) |
| **18. Wrong sex: abort?** | **Yes** | 0.7 (3) | 0.5 (1) | 0.5 (1) | 0.7 (3) | 0.7 (3) | 0.5 (1) | 0.7 (3) | 0.7 (1) | 0 (0) |
|  | **No** | 99.3 (437) | 99.5 (1) | 99.5 (216) | 99.3 (412) | 99.3 (438) | 99.5 (201) | 99.3 (444) | 99.3 (134) | 100 (60) |
| **19. US useful?** | **Yes** | 98.0 (431) | 98.5 (200) | 98.2 (213) | 98.1 (407) | 98.2 (433) | 98.0 (198) | 97.5 (436) | 99.3 (134) | 100 (60) |
|  | **No** | 1.6 (7) | 1.5 (3) | 1.4 (3) | 1.7 (7) | 1.6 (7) | 1.5 (3) | 2.0 (9) | 0.7 (1) | 0 (0) |
| **20. Pay money?** | **Yes** | 24.5 (108) | 30.5 (62) | 20.3 (44) | 29.4 (122) | 27.0 (119) | 25.2 (51) | 24.8 (111) | 36.3 (49) | 16.7 (10) |
|  | **No** | (75.2) 331 | 69.5 (141) | 79.7 (173) | 70.4 (292) | 73.0 (322) | 74.3 (150) | 74.9 (335) | 65.2 (88) | 83.3 (50) |
| **21. Continue?** | **Yes** | 98.9 (435) | 99.0 (201) | 98.6 (214) | 99.0 (411) | 99.3 (438) | 98.0 (198) | 99.3 (440) | 100 (135) | 100 (60) |
|  | **No** | 0.9 (4) | 0.0 (0) | 0.9 (2) | 0.5 (2) | 0.5 (2) | 1.0 (2) | 0.9 (4) | 0 (0) | 0 (0) |

Data shown as % (n).

The question number refers to the questionnaire (Supporting file S1). Abbreviations: N number, US ultrasound
